# Protective effect of tertiary lymphoid structures against hepatocellular carcinoma: New findings from a genetic perspective

**DOI:** 10.3389/fimmu.2022.1007426

**Published:** 2022-09-14

**Authors:** Weili Jia, Qianyun Yao, Yanfang Wang, Zhenzhen Mao, Tianchen Zhang, Jianhui Li, Ye Nie, Xinjun Lei, Wen Shi, Wenjie Song

**Affiliations:** ^1^ Xi'an Medical University, Xi’an, China; ^2^ Department of Hepatobiliary Surgery, Xijing Hospital, Fourth Military Medical University, Xi’an, China; ^3^ Department of General Surgery, The First Affiliated Hospital of Anhui Medical University, Hefei, China

**Keywords:** tertiary lymphoid structures (TLS), hepatocellular carcinoma (HCC), immunotherapy, cancer prognosis, immune microenvironment (IME)

## Abstract

**Background:**

Tertiary lymphoid structures (TLS) have an effect on hepatocellular carcinoma (HCC), but the underlying mechanism remains to be elucidated.

**Methods:**

Intratumoral TLS (iTLS) was classified in the Cancer Genome Atlas-Liver Hepatocellular Carcinoma (TCGA-LIHC) cohort using pathological sections from the Cancer Digital Slide Archive. Univariate and multivariate Cox regression analyses were performed to validate the effect of iTLS on overall survival (OS), relapse-free survival (RFS), and disease-free survival (DFS). The genes differentially expressed between the iTLS-negative and iTLS-positive groups were analyzed in combination with sequencing data. Gene set enrichment analysis (GSEA) was used to explore the signaling pathways affected by these differentially expressed genes. The random forest algorithm was used to identify genes with the highest correlation with the iTLS in the training set. Multivariate logistic regression was used to build a model to predict iTLS in tissue samples. Spearman’s correlation was used to analyze the relationship between TLS-associated chemokines and signature genes, and CIBERSORT was used to calculate immune infiltration scores. Copy number variation and its relationship with immune cell infiltration and signature genes were assessed using the gene set cancer analysis (GSCA). The Correlation R package was used for gene ontology (GO), disease ontology (DO), and gene mutation analyses. The GSCA was used for drug sensitivity analysis. LASSO regression was used to build prognostic models, and external data were used to validate the models.

**Results:**

There were 218 positive and 146 negative samples for iTLS. iTLS was significantly associated with better RFS and DFS according to Cox regression analysis. Twenty signature genes that were highly associated with iTLS positivity were identified. GO and mutation analyses revealed that the signature genes were associated with immunity. Most signature genes were sensitive to immune checkpoint inhibitors. Risk scores calculated using a characteristic gene-based prognostic model were found to be an independent prognostic factor for OS.

**Conclusions:**

The improvement of RFS in HCC by iTLS was not limited to the early period as previously reported. iTLS improved DFS in patients. Characteristic genes are closely related to the formation of iTLS and TLS chemokines in HCC. These genes are closely related to immunity in terms of cellular infiltration, biological functions, and signaling pathways. Most are sensitive to immune checkpoint inhibitors, and their expression levels can affect prognosis.

## 1 Introduction

As of 2020, hepatocellular carcinoma (HCC) was the sixth leading cause of cancer-related deaths, making it one of the world’s leading public health problems (1). The incidence of HCC continues to increase annually. According to World Health Organization estimates, by 2040 the number of new cases and deaths will exceed 1.4 million and 1.3 million, respectively (2). Tertiary lymphoid structures (TLS), also known as tertiary lymphoid organs (TLO) and ectopic lymphoid structures (ELS), are aggregates of lymphocytes capable of providing ectopic hubs for the acquired immune response, and can affect various disease outcomes (3). TLS is acquired and often formed under the stimulation of chronic inflammation to address the invasion of various pathogenic factors (4). The first study on TLS was conducted on non-small-cell lung cancer (NSCLC). Seventy-four patients were studied using immunohistochemistry in the early stages (5). Subsequently, the amount of TLS-related literature has increased annually, and more scientists are involved in the research of TLS every year.

Most patients with HCC are not diagnosed until in the advanced stages, thus missing the optimal window for treatment (6). The insensitivity of HCC to conventional malignancy therapies has led to the emergence of immunotherapy as one of the most promising treatments (7). As an immune structure, TLS is gaining attention from researchers worldwide. There is growing evidence of its direct and indirect impact on HCC outcomes. While TLS in cancer is generally protective, two contrasting effects have been reported in HCC: intratumoral TLS (iTLS) can be protective against HCC, while peritumoral TLS (pTLS) can be detrimental for HCC. iTLS may be associated with sustained and effective anti-tumor immunity (8–12). Additionally, more mature iTLS could help improve patient prognosis (9). Conversely, there is evidence that pTLS can promote HCC development (13, 14). TLS found in excised non-neoplastic liver tissue surrounding HCC is associated with poor prognosis and increased prevalence (9, 14). In addition, studies have shown that depletion of TLS in non-neoplastic liver parenchyma can inhibit cancer progression (14). However, a recent study showed that patients with HCC and higher pTLS densities had better overall survival (OS) and relapse-free survival (RFS) (15).

Few studies have analyzed TLS at the genetic level, particularly in HCC. Therefore, we performed a bioinformatic analysis in combination with hematoxylin and eosin (HE) pathologically stained sections to explore and elucidate the genetic characteristics of iTLS in HCC and the prognostic implications of differential expression of genes related to iTLS in patients. Together with previous studies, we believe that our study will contribute to further understanding of the role of iTLS in HCC.

## 2 Materials and methods

### 2.1 Data acquisition and processing

XENA (https://xena.ucsc.edu) is an online discovery tool for public and private multi-omics and clinical/phenotypic data (16). We used this tool to obtain the liver hepatocellular carcinoma (LIHC) dataset from The Cancer Genome Atlas (TCGA) in the RNAseq count format. The data were processed as follows: 1) data from the same sample but from different tables were averaged; 2) data from different samples were combined to form a genomic matrix; and 3) a log2(x+1) transformation was performed on all data. Data from the International Cancer Genome Consortium (ICGC) cohort were obtained from the official website. We downloaded the LIRI-JP data from the ICGC along with the corresponding clinical information of the cohort. Please refer to **Supplementary Table 1** for a summary of the relevant data.

### 2.2 Evaluation of iTLS in pathological sections

We evaluated the density of lymphocyte infiltration by retrieving HE pathologically stained sections of the corresponding TCGA samples from the Cancer Digital Slide Archive (CDSA). Frozen sections and formalin-fixed paraffin-embedded (FFPE) tissue sections were used. This study used methods based on Clarice et al. (17) for counting all forms of iTLS as follows: 1) lymphocyte aggregates (Agg) with lymphocyte infiltration but no lymphoid follicle formation; 2) primary follicles (FL1), with well-defined clusters of round or oval lymphocytes or plasma cells (no germinal centers present); and 3) secondary follicles (FL2), with well-defined clusters of round or oval lymphocytes or plasma cells (germinal centers present). Based on the above groupings, two pathologists independently evaluated iTLS in all HCC samples. Subsequently, a third pathologist assisted in identifying the conflicting results. Then, based on the technique of Clarice et al. (17), samples with at least one occurrence of any form of iTLS (Agg, FL1, FL2) were categorized as the TLS-positive (TLS+) group and the samples without any occurrence of iTLS were categorized as the TLS-negative (TLS-) group for the next analysis.

### 2.3 Analysis of the impact of iTLS on the prognosis of HCC

Previously, Li et al. (11) and Calderaro et al. (8) found the significance of iTLS in HCC for better early stage RFS in patients, but further validation was needed to support this conclusion. Therefore, to verify their findings or to discover new information, we plotted the iTLS grouping status (positive or negative) in relation to OS, RFS, and disease-free survival (DFS) to determine the prognostic impact of iTLS after excluding patients with incomplete follow-up information. OS was defined as the time from the start of follow-up until the patient died or was lost to follow-up for any reason, RFS was defined as the time from the start of follow-up until the patient experienced a disease recurrence, and DFS was defined as the time from the start of follow-up until the patient died, was lost to follow-up, or experienced a disease recurrence for any reason. Univariate and multivariate Cox regression analyses were performed to determine whether the iTLS was an independent prognostic factor. Indicators with significant results (p < 0.05) in the univariate analysis were included in the multivariate analysis.

### 2.4 Analysis of gene expression differences

To investigate the differential gene expression between the two groups, we selected intra-tumor pathology samples with both HE stained sections and RNAseq expression data, and then performed differential expression analysis using the R package “edgeR” according to the TLS grouping (TLS+ or TLS-). First, samples with zero expression were excluded. Differential expression analysis was performed to explore differences between the two groups. Finally, gene set enrichment analysis (GSEA) was performed using the KEGG and Reactome databases to clarify the signaling pathways in the locations of the differential genes.

### 2.5 Identification of key genes associated with iTLS

The Boruta algorithm was used to identify the key differential genes affecting iTLS formation. Boruta is a feature selection algorithm, which is specifically a wrapper algorithm for random forests that filters out the set of all features correlated with the dependent variable (18). It can manage a large number of input variables, evaluate the importance of the variables during processing, and has been used in many studies in cancer-causative gene analysis (19, 20). To explore all potential genes that may have an impact on the formation of iTLS, we included all genes in which differential expression analysis showed significant results (p < 0.05) in the subsequent study.

We first used the Boruta algorithm to rank the differential genes from largest to smallest according to their effects on iTLS. The top 20 results were selected as iTLS signature genes, which were later used to build a logistic regression model for predicting the presence of iTLS. The specific steps were as follows: 1) the samples were randomly divided into training and test groups; the training group was used to select the key genes affecting the formation of iTLS and the test group was used to validate the results from the training group; 2) all differential gene expressions in the training group were characterized using the R package “Boruta” to identify the key categorical variables; 3) five 10-fold crossover validations were performed using the R package “caret” to find the most accurate mtry value (mtry refers to the number of variables randomly sampled when constructing decision tree branches in random forest modeling, and an appropriate mtry value can reduce the prediction error rate of the random forest model); and 4) the final selected model was extracted and receiver operating characteristic (ROC) curves were created using the validation set data to verify the prediction ability of the model situation to establish a multivariate logistic regression model. ROC curves were plotted for all samples to further validate the predictive ability of the model. Previous studies used several genetic traits relevant to TLS to assess TLS in tissues. Results showed that these traits had different combinations, such as the 9-TLS trait (21), 12-TLS trait (22), 40-TLS trait (23), and 50-TLS trait (24). We selected these chemokines, developed correlation prediction models, and compared them with our model.

### 2.6 Immune cell infiltration analysis

iTLS belongs to the immune structure family. Therefore, one can hypothesize that there is a correlation between iTLS and immune cell infiltration. Hence, we calculated the immune infiltration score for each patient’s cancer tissue using CIBERSORT. The relationship between the signature genes and the immune infiltration score was then analyzed using Spearman correlation analysis. Finally, the effect of copy number variation (CNV) on immune cell infiltration was explored using gene set cancer analysis (GSCA).

### 2.7 Biological functional analysis

The R package “clusterProfiler” supports the functional characterization of thousands of coding and non-coding genomic data with up-to-date gene annotations (25). It provides a unified interface for gene function annotations from a variety of sources and can therefore be applied to a variety of scenarios. We used this program for gene ontology (GO) biological processes, GO cellular composition, GO molecular function, and disease ontology (DO) analysis.

### 2.8 Single nucleotide polymorphism (SNP) and CNV analysis

To understand the mutation of signature genes within tumors, we investigated single nucleotide polymorphism (SNP) mutations in signature genes using the R package “maftools”, while exploring the signaling pathways affected by the mutations. In addition, using GSCALite, a web-based platform for genomic cancer analysis (26), we investigated the CNV of the signature genes.

### 2.9 Drug sensitivity analysis

The ultimate goal of medical research is to facilitate clinical treatment; therefore, we explored the relationship between signature genes and drug sensitivity using GSCA. The tool contains data from both the GDSC and Clinical Trials Reporting Program (CTRP) databases. GDSC (27) characterized 1000 human cancer cell lines and screened them for more than 100 compounds; CTRP (28) has similar characterization and screening of data. We further downloaded gene expression and drug sensitivity data from CellMiner, a web-based suite of genomics and pharmacology tools (29). Subsequently, drug sensitivity data were screened by selecting drugs that were validated by clinical trials and Food and Drug Administration (FDA) approval. Finally, the expression data of the characterized genes were subjected to the Spearman correlation test with the drug sensitivity data to obtain and visualize the correlation data between them.

### 2.10 Construction and validation of prognostic model

To explore the prognostic value of signature genes, LASSO regression was used to further screen genes from the signature genes that clearly affect the prognosis of HCC. The optimal signature model was then constructed based on the Akira pooling information criterion (AIC), and the result with the lowest AIC value was used to construct the signature: risk score = expression(A) × c of (A) + expression(B) × c of (B) +. expression(n) × c of(n). OS, DFS, and RFS curves were plotted based on risk scores using the Kaplan-Meier method. The accuracy of the model was verified using ROC curves. Univariate and multivariate Cox regression analyses were used to identify the model as an independent influence on prognosis, and heatmaps of the risk score, relevant clinical indicators, and characteristic gene expression were plotted. Finally, we validated the predictive power of the model using the ICGC data as external data.

### 2.11 Statistical analysis

All data analysis, data visualization (graphical plots), and statistical analysis were performed using R Studio Desktop (version 4.1.2), unless otherwise specified. Logistic regression analysis was performed using the R software. Gene difference analysis was performed using R package “edgeR”. The R package “GSVA” was used for the ssGSEA analysis. Correlation analysis was performed using the R package “Hmisc”. SNP mutation analysis was performed using the R package “maftools”. The R package “Boruta” was used for random forest analysis. Survival analysis was performed using the R package “survivor”. The R package “survivalROC” was used to plot the time-dependent ROC curves. RFS analysis was performed using Kaplan-Meier plots and log-rank tests. Correlations between the two non-normal datasets were analyzed using Spearman’s method. The Wilcoxon signed-rank test was used to evaluate between-group differences in pathological parameters. The cardinality test was used to analyze the relationship between the clinicopathological parameters and characteristics. P values below 0.05 were considered statistically significant if not otherwise stated.

## 3 Results

### 3.1 iTLS is associated with better RFS and DFS in patients

**Figure 1:**
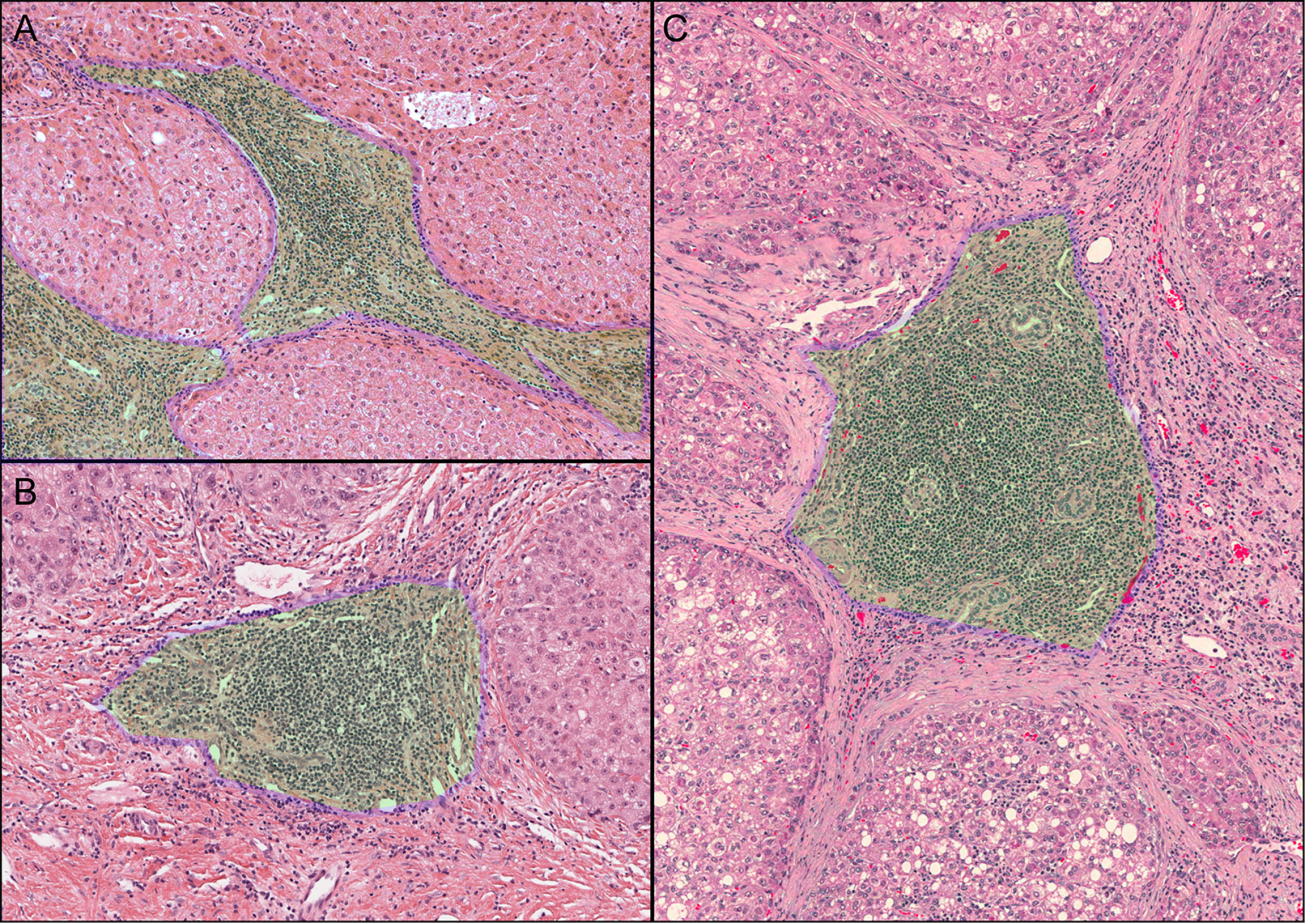
Observed iTLS images. The green area with the blue border marked in the figure is the iTLS. **(A)** Lymphocyte aggregation (Agg). **(B)** Primary lymphoid follicles (FL1). **(C)** Secondary lymphoid follicles (FL2).

**Figure 2:**
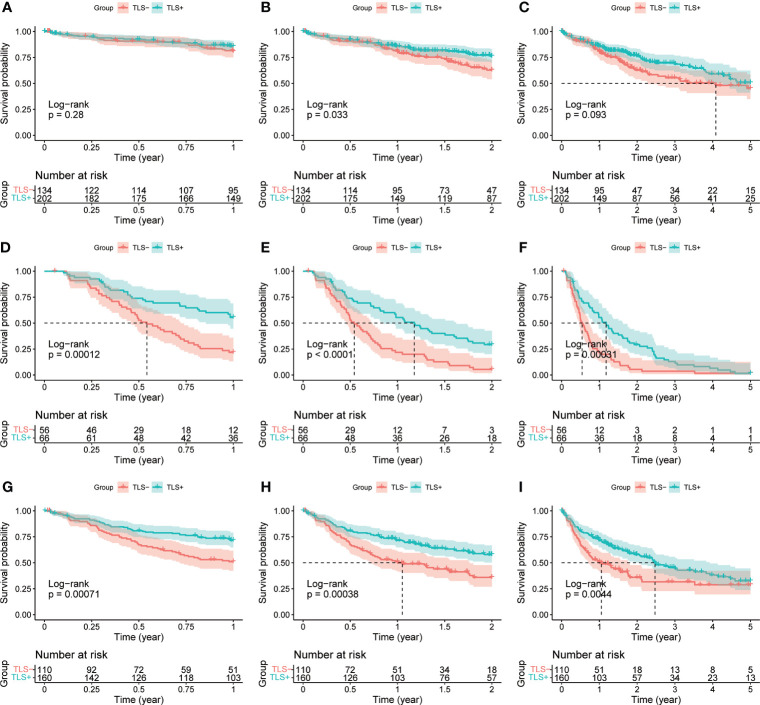
**(A)** 1-year OS curves of TLS-group vs. TLS+ group. **(B)** 2-year OS curves of TLS-group vs. TLS+ group. **(C)** 5-year OS curves of TLS-group vs. TLS+ group. **(D)** 1-year RFS curves of the TLS-group versus the TLS+ group. **(E)** 2-year RFS curves of the TLS-group versus the TLS+ group. **(F)** 5-year RFS curves of the TLS-group versus the TLS+ group. **(G)** 1-year DFS curves of the TLS-group versus the TLS+ group. **(H)** 2-year DFS curves of TLS-group vs. TLS+ group. **(I)** 5-year DFS curves of TLS-group vs. TLS+ group.

A total of 365 samples were observed; 137 samples were classified as the Agg group, 56 as the FL1 group, and 26 as the FL2 group. The remaining 146 samples had no lymphocytic infiltration (**Figure 1**). There were 218 samples in the final TLS+ group and 146 in the TLS- group (**Supplementary Table 2**). Results of OS, RFS, and DFS curves showed that the TLS+ group was associated with better RFS (p-values less than 0.001 at 1, 2, and 5 years), DFS (p<0.001, p<0.001, p = 0.004 at 1, 2, and 5 years, respectively), and 2-year OS (p = 0.033) (**Figure 2**).

**Figure 3:**
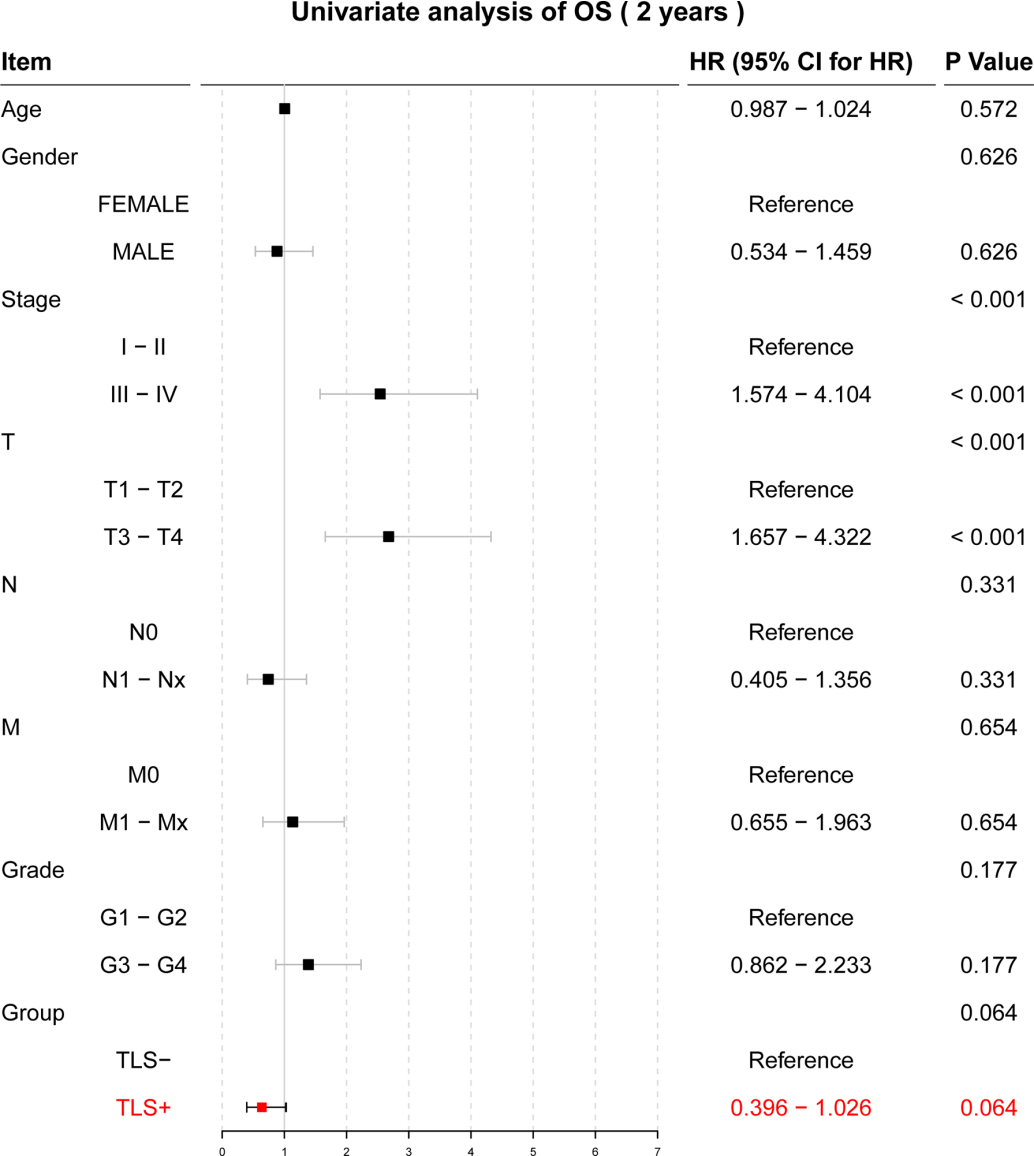
Results of the univariate Cox regression analysis with two-year OS as the outcome.

**Figure 4:**
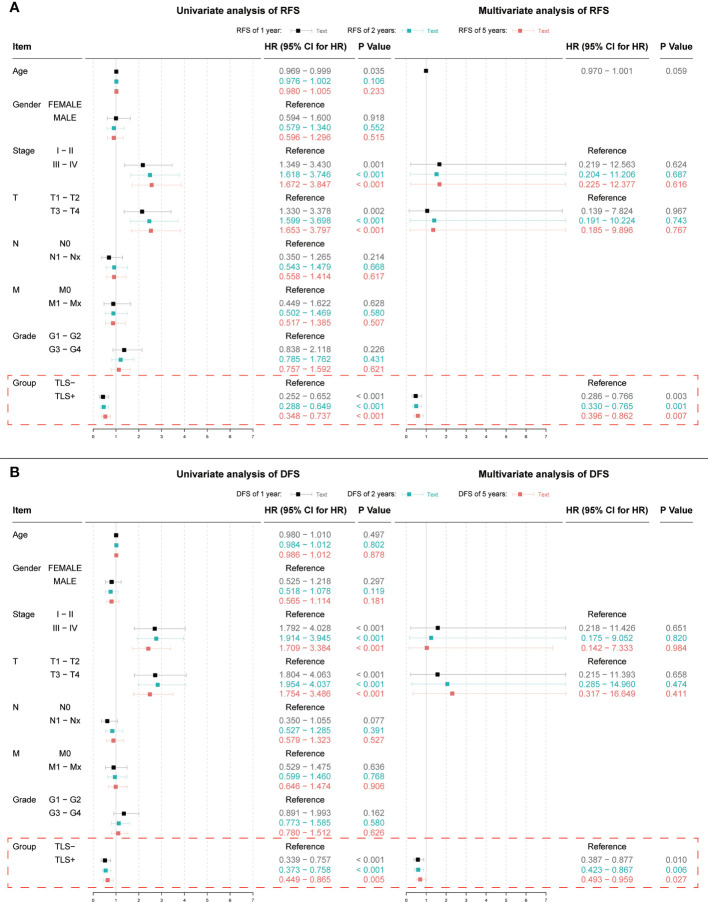
**(A)** Results of univariate and multivariate Cox regression analyses with 1, 2, and 5-year RFS as outcomes; **(B)** Results of univariate and multivariate Cox regression analyses with 1, 2, and 5-year DFS as outcomes. Indicators with significant (p < 0.05) results in the univariate Cox regression analysis are further included in the multivariate Cox regression analysis.

Univariate Cox regression results for OS were not statistically significant (p = 0.064, **Figure 3**). Univariate and multivariate Cox regression results for both RFS and DFS showed that iTLS (TLS+) was a protective factor (**Figure 4**). Interestingly, in a slight departure from the results of previous studies (8, 11), we found that iTLS was not only associated with early RFS. Rather, at all periods (1, 2, and 5 years), iTLS was associated with better RFS. This further validates the beneficial effects of iTLS in patients with HCC.

### 3.2 Differential genes are associated with immune-dominated pathways

The results of the differential expression analysis showed that of the 1057 differential genes, most (625 genes) were downregulated, whereas the expression of the other 432 showed varying degrees of upregulation (**Supplementary Table 3**).

**Figure 5:**
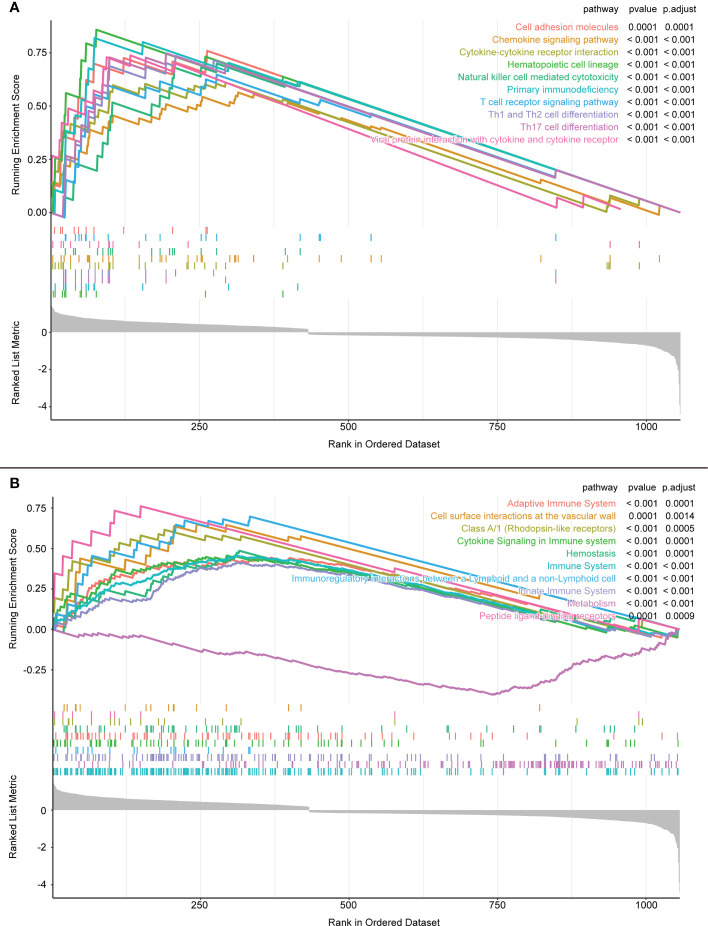
**(A)** GSEA enrichment analysis results of KEGG database. **(B)** GSEA enrichment analysis results of Reactome database.

In the GSEA, the KEGG database showed that the differential genes were mainly distributed in “cell adhesion molecules”, “chemokine signaling pathway”, and “cytokine-cytokine receptor interaction” pathways (**Figure 5A**). Conversely, the Reactome database reported that the differentially expressed genes were mainly enriched in the “Adaptive Immune System”, “Class A/l (Rhodopsin-like receptors)”, and “Cytokine Signaling in Immune System” pathways. Clearly, most of these pathways are closely related to immunity (**Figure 5B**).

### 3.3 iTLS prediction model consisting of 20 genes

**Figure 6:**
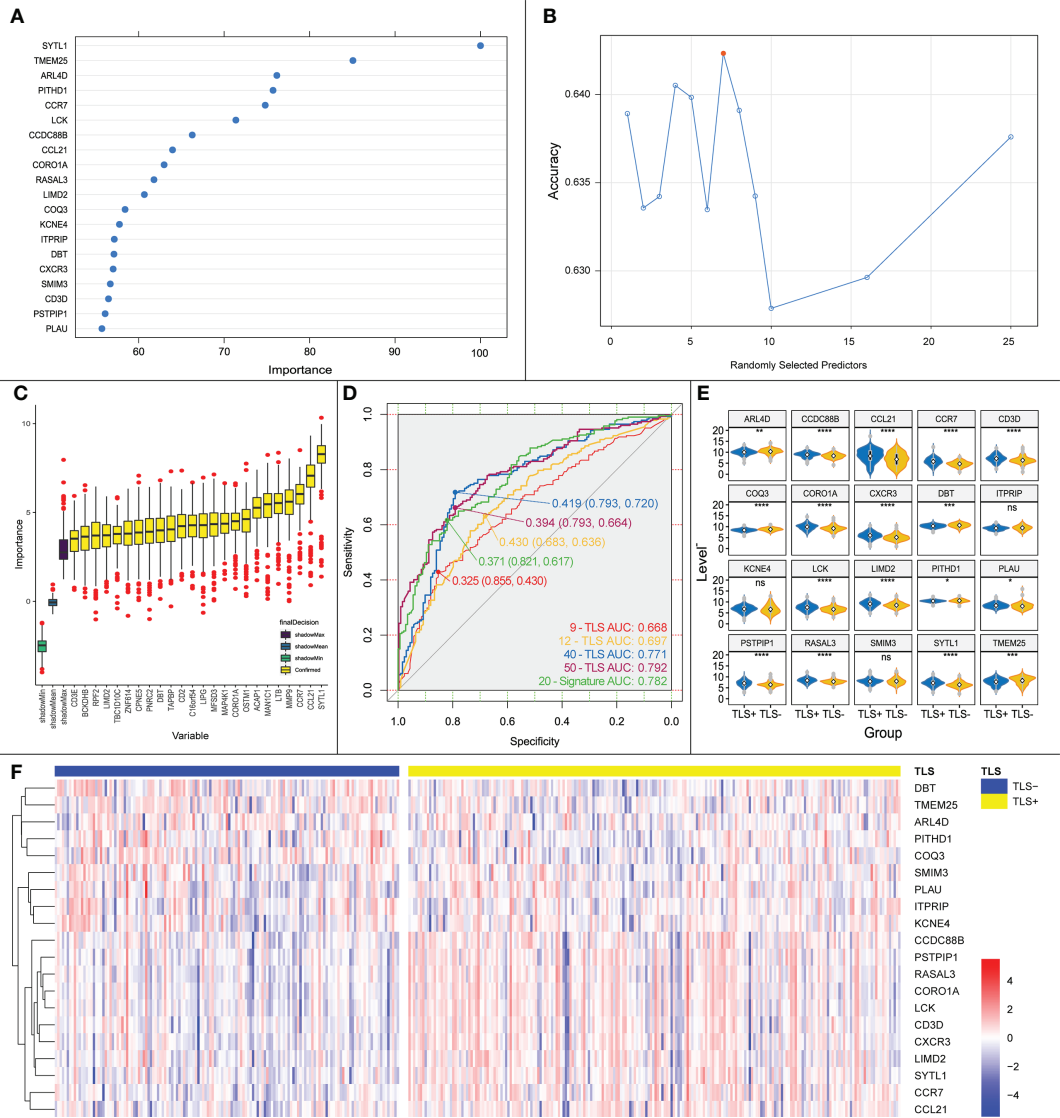
**(A)** Key genes influencing the presence or absence of iTLS screened by Boruta algorithm, horizontal coordinates are genes and vertical coordinates are the importance of the genes. **(B)** Schematic diagram of the screening process for mtry values, red points are the most appropriate values; horizontal coordinates are mtry values and vertical coordinates are the accuracy of the value. **(C)** Genes screened by Boruta’s algorithm with the relative importance of the gene in the horizontal coordinate and the gene in the vertical coordinate. **(D)** ROC curves plotted using our proposed prediction model together with the previously reported TLS chemokines. **(E)** Expression of the 20 TLS signature genes obtained by screening in each group. Significance marks, ns: p > 0.05; *:p <= 0.05; **:p <= 0.01; ***:p <= 0.001; ****:p <= 0.0001. **(F)** Heatmap based on the expression of the 20 TLS signature genes obtained by screening in each sample, from which it can be seen that with CCDC88B as demarcation, the upper genes are up-regulated in expression in the TLS+ group, and the lower genes are the opposite.

Using Boruta, we selected the most suitable mtry values (**Figure 6A**) and identified 24 important genes associated with iTLS (**Figure 6B**). A 10-fold-5 cross-validation was then performed on the training set, and the top 20 most important data points affecting the iTLS profile in the training set were selected to build the prediction model (**Figure 6C**). These 20 genes were: SYTL1, TMEM25, ARL4D, PITHD1, CCR7, LCK, CCDC88B, CCL21, CORO1A, RASAL3, LIMD2, COQ3, KCNE4, ITPRIP, DBT, CXCR3, SMIM3, CD3D, PSTPIP1, and PLAU.

The area under the curve (AUC) of the ROC curve obtained using the model in the validation set was 0.733 (**Figure 6D**). The AUC of the ROC curve of the iTLS prediction model built using multifactorial logistic regression was 0.782 for all samples (**Figure 6E**). According to the TLS grouping (positive or negative), we plotted a gene expression heatmap of the characteristic genes in all HCC samples (**Figure 6F**). As the figure shows, with CCDC88B as the dividing line, the upper genes were concentrated in the TLS+ group with high expression, whereas the lower genes were concentrated in the TLS- group.

We also built prediction models using TLS-related chemokines to verify the accuracy of our models. The results showed that the AUCs of the 9-TLS, 12-TLS, 40-TLS, and 50-TLS prediction models were 0.668, 0.697, 0.771, and 0.792, respectively. The accuracy of our results is slightly higher than that of the 40-TLS and slightly lower than that of the 50-TLS. However, one problem that cannot be ignored is that when using the 40-TLS and 50-TLS features for logistic regression, the number of independent variables is too large. Therefore, the results may not be accurate. However, considering the above factors, the predictive ability of our model remains excellent.

### 3.4 Further exploration around signature genes

#### 3.4.1 Signature genes are associated with TLS-associated chemokines

**Figure 7:**
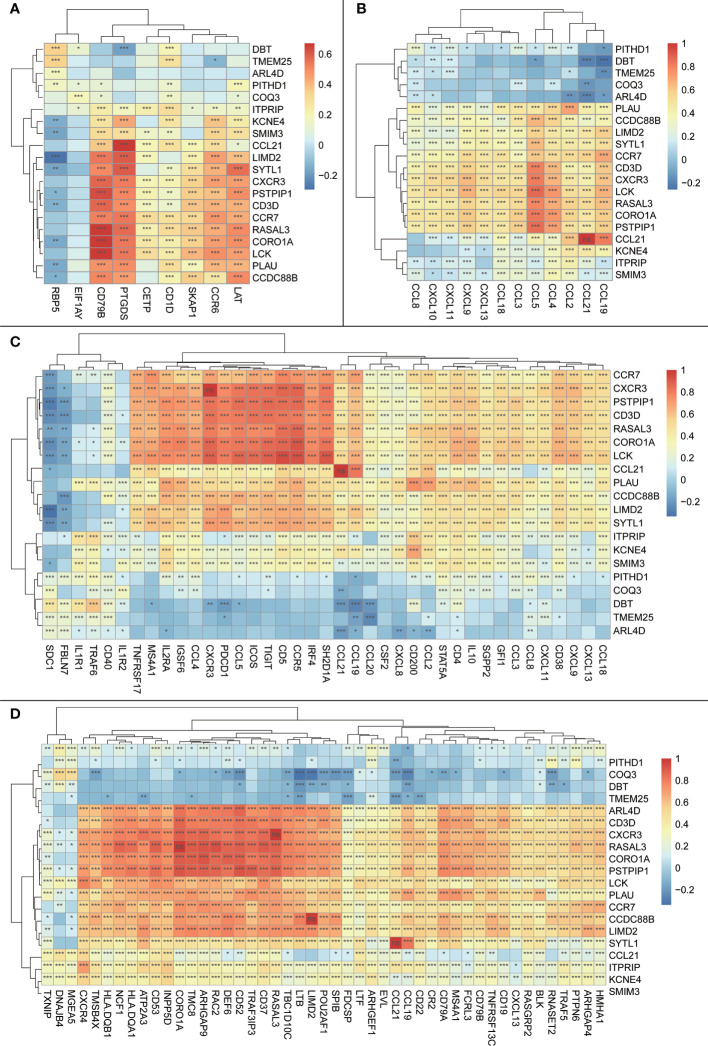
Heatmap of correlations between the trait genes and other previously reported TLS features; the vertical axis is the signature genes obtained in the current study, and the horizontal axis is other previously reported TLS signature genes; the magnitude of correlations is indicated by the block color; significance is shown in the block by symbols: ns: p > 0.05 or the two items tested for correlation are the same item; *:p <= 0.05; **:p <= 0.01. **(A)** Heatmap of correlations between trait genes and 9-TLS traits. **(B)** Heatmap of correlations between trait genes and 12-TLS traits. **(C)** Heatmap of correlation between the trait genes and 40-TLS traits. **(D)** Heatmap of correlations between the trait genes and 50-TLS features.

The Spearman correlation analysis using previously reported expression levels and the expression levels of our signature genes was performed to further validate our signature genes. The results showed that most of our signature genes had strong correlations with the aforementioned features, whether it was the classical 12-TLS feature or the 9-TLS feature proposed by Feng et al. (21), the 40-TLS feature proposed by Zhou et al. (23), or the 50-TLS feature used by Wu et al. (24) (**Figure 7**).

#### 3.4.2 Signature genes are associated with immune infiltration

**Figure 8:**
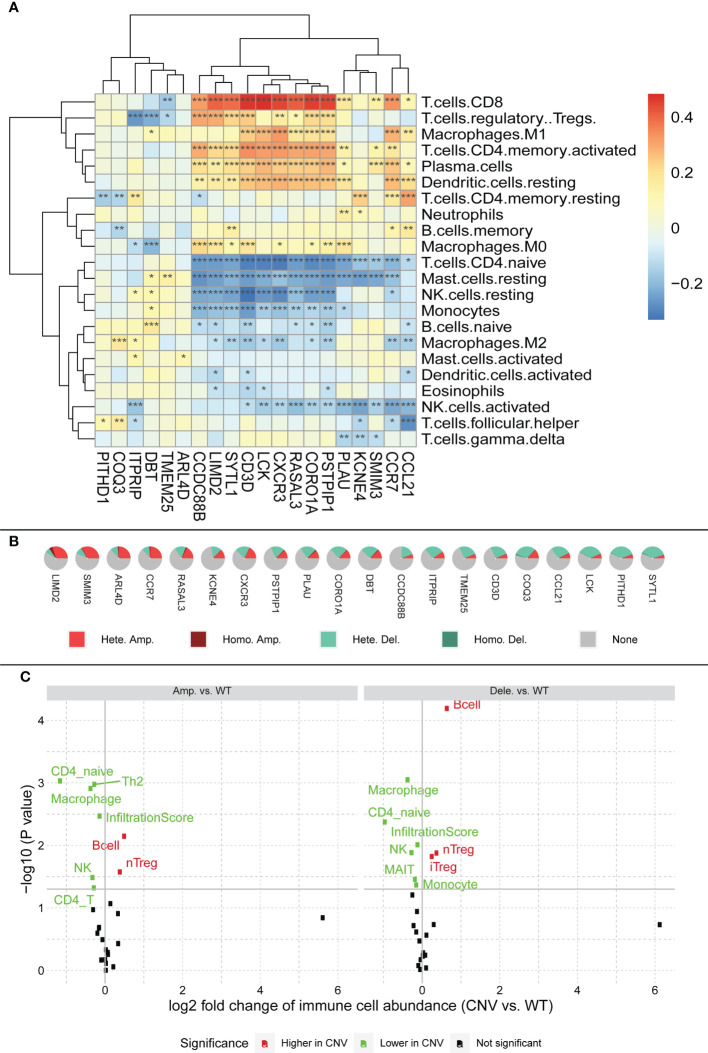
**(A)** Relationship between characteristic genes and immune cell infiltration, block colors represent the level of correlation. Significance markers: *p <= 0.05; **p <= 0.01; ***p <= 0.001. **(B)** CNV profile of feature genes in HCC; legend meanings are as follows: Hete amp, the percentage of samples with copy number heterozygous amplification; Hete dele, the percentage of samples with copy number heterozygous deletion; Homo amp, the percentage of samples with copy number homozygous amplification; Homo dele, the percentage of samples with copy number homozygous deletion. **(C)** Relationship between CNV and immune cell infiltration; black dots in the figure represent no effect of CNV on immune cell infiltration, red dots represent enhanced immune cell infiltration caused by CNV, and green dots represent diminished immune cell infiltration caused by CNV.

We plotted a heatmap of the correlation between the expression of the signature gene and calculated the immune score using CIBERSORT (**Supplementary Table 4** and **Figure 8A**). Using CD4 T cells as the boundary, the level of immune cell infiltration above the boundary is roughly positively correlated with the signature gene, whereas the level of immune cell infiltration below the boundary is roughly negatively correlated with the signature gene (**Figure 8A**).

Serendipitously, using the GSCA (http://bioinfo.life.hust.edu.cn/GSCA/), we found a large number of CNV in the signature genes, most of which were heterozygous variants (**Figure 8B**). Among them, LIMD2 had the most heterozygous amplifications, whereas SYTL1 had the most heterozygous deletions. Based on the relationship between signature genes and immunity, we hypothesized that the CNV of signature genes might affect immune infiltration. Therefore, we explored the relationship between CNV and immune cell infiltration (**Figure 8C**). The figure shows that both gene amplification and deletion increase B-cell infiltration, whereas the infiltration levels of CD4_naïve, NK, and Macrophage are all diminished. The figure also shows that both amplification and deletion mutations cause a decrease in the immune infiltration score, which in turn affects immune infiltration (see infiltration score in **Figure 8C**).

In addition, we used the immune database ImmPort1 for further analysis to explore the relationship between signature genes and immune genes. The results showed that most of the immune genes correlated with our signature genes, with the strongest correlations being ARL4D and CCL (**Supplementary Table 5** and **Supplementary Figure 1**).

#### 3.4.3 Signature genes are associated with multiple biological functions and hepatobiliary diseases

**Figure 9:**
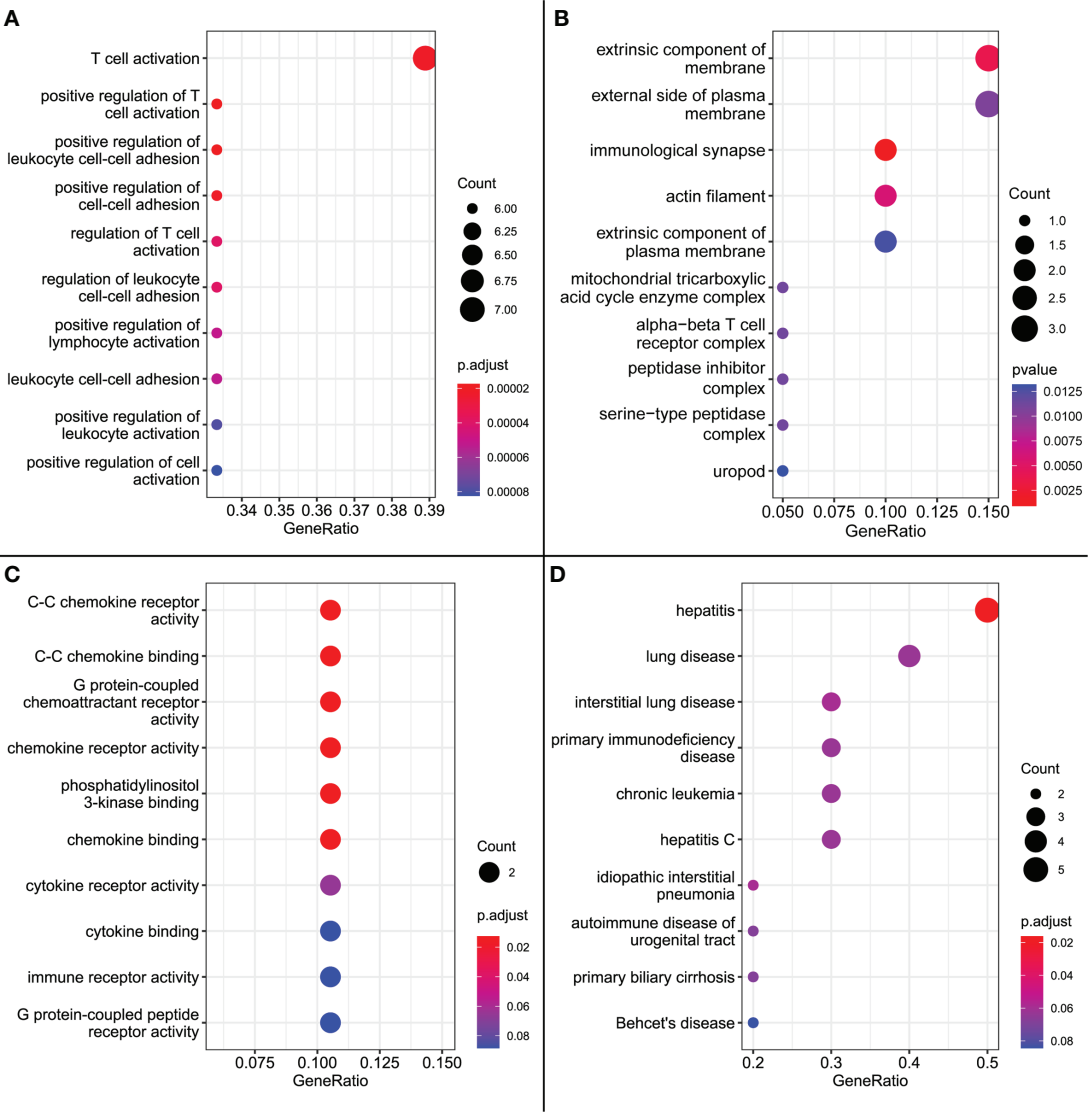
**(A)** GO enrichment results for biological processes. **(B)** GO enrichment results for cellular composition. **(C)** GO enrichment results for molecular functions. **(D)** DO enrichment results.

GO enrichment analysis showed that, in terms of biological processes, the 20 signature genes were significantly associated with immune processes including “T cell activation”, “positive regulation of T cell activation”, and “positive regulation of leukocyte cell adhesion” (p < 0.001). Additionally, cell composition was associated with “immunological synapse”, and molecular function was associated with “C-C chemokine receptor activity”, “C-C chemokine binding”, and “G protein-coupled chemoattractant receptor activity” (p = 0.02). The results of DO analysis were also associated with “hepatitis”, “hepatitis C”, and “primary biliary cirrhosis” (**Figure 9**).

#### 3.4.4 Mutations in signature genes can affect cancer-related signaling pathways

**Figure 10:**
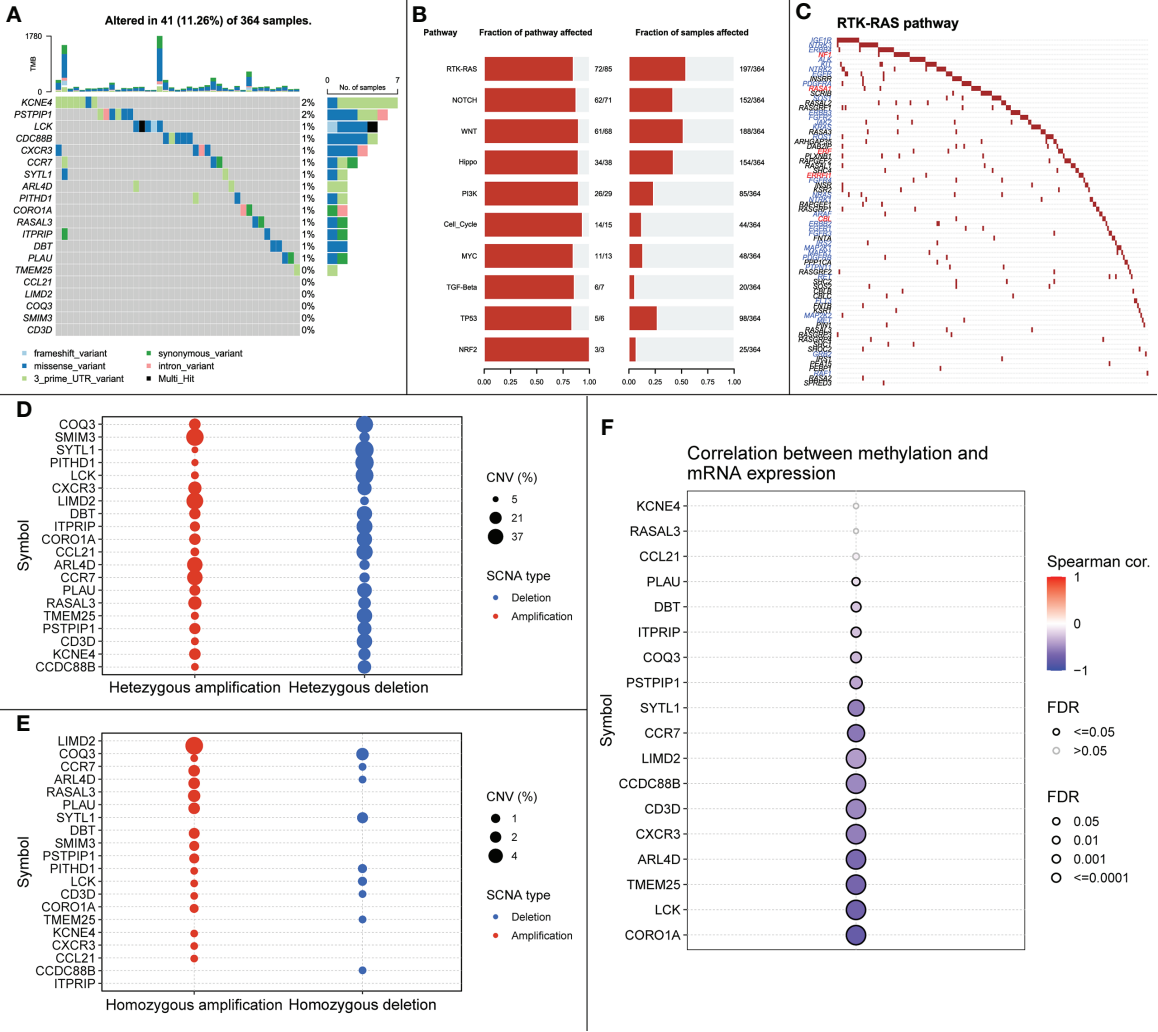
**(A)** SNP mutations in all samples. **(B)** Signaling pathways affected by feature genes in all samples. **(C)** Mutations in RTK-RAS pathway in all samples, blue font represents cancer-promoting mutations, red font represents cancer-suppressing mutations. **(D)** Heterozygous CNV mutations in the TCGA-LIHC cohort for the signature gene. **(E)** Pure-zygous CNV mutations in the TCGA-LIHC cohort for the signature gene. **(F)** Relationship between mRNA expression of the signature gene and methylation.

SNP, mainly DNA sequence diversity caused by variants in a single nucleotide at the genomic level, can lead to the development of disease. We further investigated the SNP profiles of the characteristic genes and mapped the mutations between samples (**Figure 10A**).

The close relationship between cancer progression and signaling pathways is well known. Therefore, we investigated the signaling pathways affected by relevant signature genes in each group of samples (**Figure 10B**). RTK-RAS, a pathway known to influence cancer progression, ranked first among the affected pathways; therefore, we mapped the RTK-RAS pathway in terms of gene mutations (**Figure 10C**). Methylation is one of the first identified and most intensively studied epigenetic regulatory mechanisms that can influence the progression of many cancers. We explored the correlation between methylation of characteristic genes and mRNA expression between normal and cancer samples. The expression of CORO1A, LCK, and ARL4D showed a significant negative correlation with methylation (**Figures 10D–F**).

**Figure 11:**
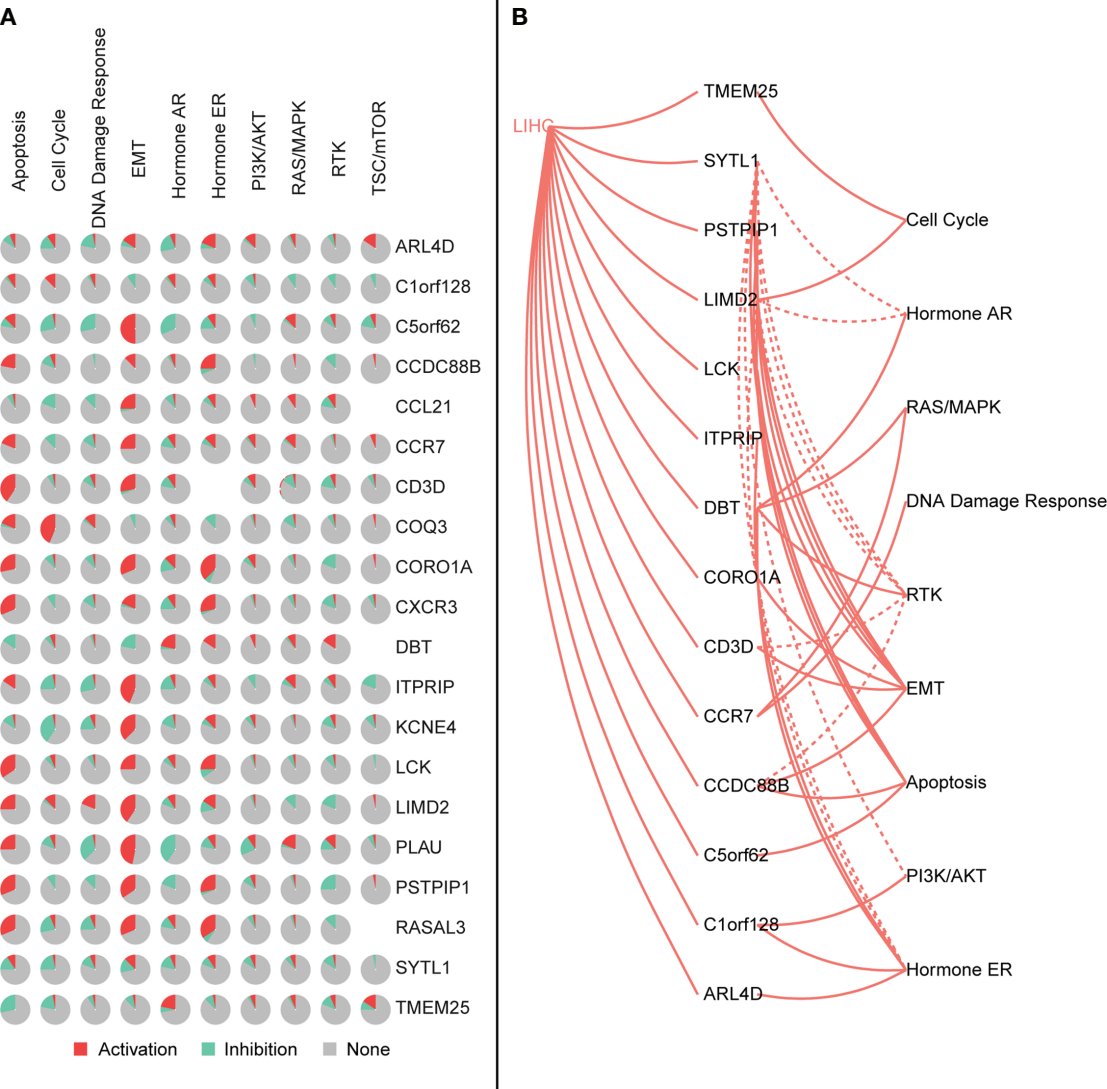
**(A)** Characteristic genes affect the activation or inhibition of the pathway, where red represents the pathway being activated and green represents the pathway being inhibited. **(B)** Interaction between the characteristic genes and the pathway, where the solid line is the pathway being activated and the dashed line is the pathway being inhibited.

We further explored the relationship between signature genes and CNV using GSCALite. We also used this tool to investigate the effect of gene expression differences on pathway activation, where all mutations affected more than one signaling pathway (**Figure 11**).

#### 3.4.5 Signature genes are sensitive to immune checkpoint inhibitors

**Figure 12:**
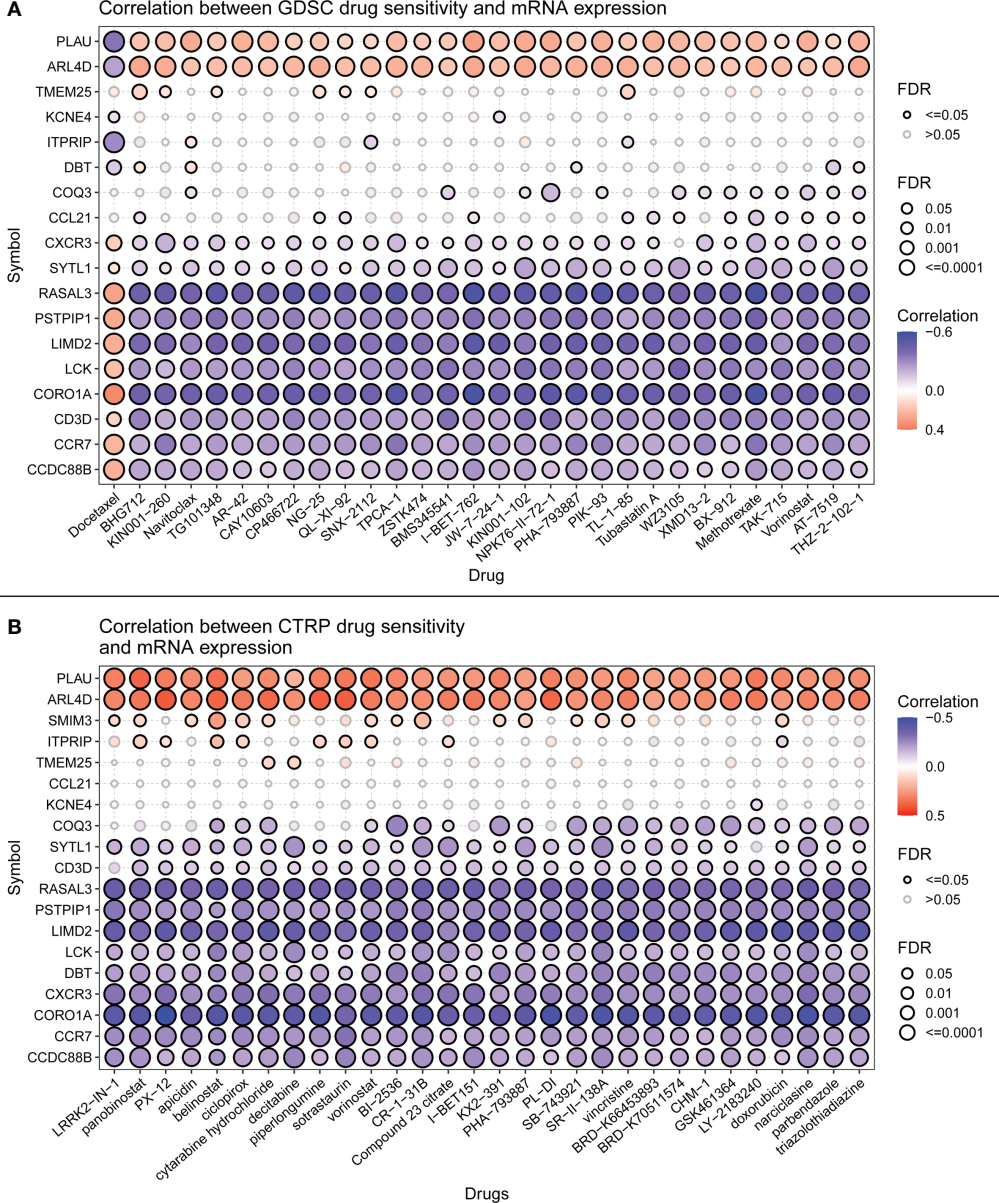
**(A)** Drug sensitivity of signature genes in GDSC. **(B)** Drug sensitivity of signature genes in CTRP.

Immune checkpoint inhibitors have been approved as conventional drugs for HCC, and the possibility of immunotherapy should be further investigated. We explored the correlation of signature genes with the sensitivity to GDSC and CTRP drugs in pancreatic cancer using the GSCA website. As seen in **Figure 12**, both the GDSC and CTRP databases showed that RASAL3 and COROIA had the highest sensitivity to immune checkpoint inhibitors, suggesting that these genes may be potential therapeutic targets. LIMD2, PSTPIP1, CD3D, LCK, and CCR7 were significantly negatively correlated with the IC50 of most drugs, whereas CCDC88B, ITPRIP, SYTL1, PLAU, TMEM25, and ARL4D were correlated with some drugs to varying degrees. PITHD1 is not presented in the figure because of a lack of relevant data.

**Figure 13:**
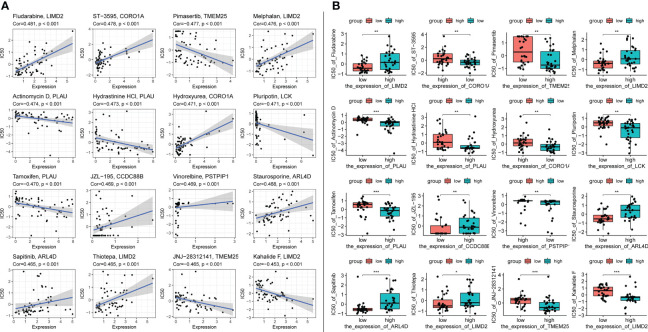
Top 16 drug effects with the highest correlation in the CellMiner database for characteristic genes, gene and drug names are identified in the figure by chart title or vertical coordinate. **(A)** Horizontal axis is gene expression, vertical axis is drug IC50. **(B)** Horizontal axis is gene expression along median dichotomous classification, vertical axis is drug IC50. significance markers, ns: p > 0.05; *p <= 0.05; **p <= 0.01; ***p <= 0.001.

Using the CellMiner database, we evaluated the relationship between signature genes and drug IC50 (**Supplementary Table 5**). From this analysis, we selected the portion of the data presented in the figure with the most significant effect (**Figure 13**). The results showed that 20 signature genes may be promising potential drug targets for HCC and merit further in-depth study.

#### 3.4.6 Signature genes have a significant impact on the prognosis of HCC

**Figure 14:**
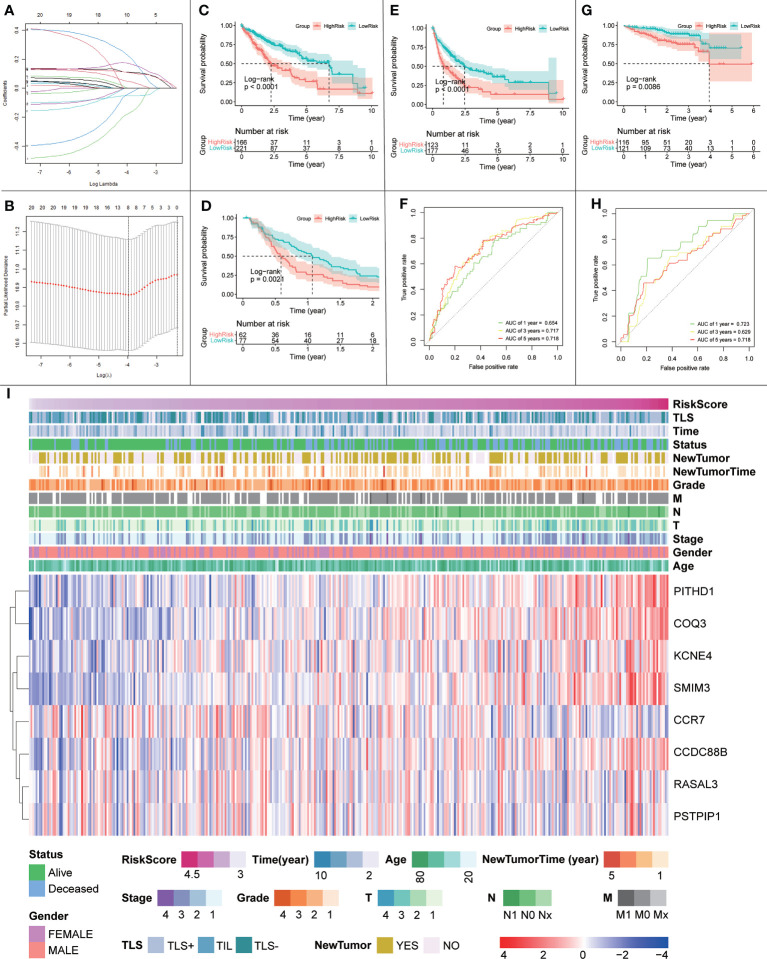
**(A, B)** Schematic diagram of the LASSO regression variable shrinkage screening process. **(C)** OS of the prognostic model in the TCGA cohort. **(D)** RFS of the prognostic model in the TCGA cohort. **(E)** DFS of the prognostic model in the TCGA cohort. **(F)** Time-dependent ROC curve of the prognostic model in the TCGA cohort. **(G)** OS curve of the prognostic model in the ICGC cohort. **(H)** Prognostic model in the ICGC cohort with time-dependent ROC curves. **(I)** Heatmap of risk scores, clinical features and expression of signature genes.

Given the protective effect of iTLS on HCC prognosis, we hypothesized that signature genes could be used to predict patient prognosis. Therefore, we used the LASSO regression to build a prognostic model based on a 20-trait gene screen (**Figures 14A, B**). Twelve genes were eliminated during the screening process and the final prognostic model was obtained as follows:

*0.109×exp(PITHD1)+-0.178×exp(RASAL3)+-0.053×exp(CCR7)+0.171×exp(COQ3)+-0.042×exp(PSTPIP1)+0.045×exp(KCNE4)+0.224×exp(CCDC88B)+0.106×exp(SMIM3)*

**Figure 15:**
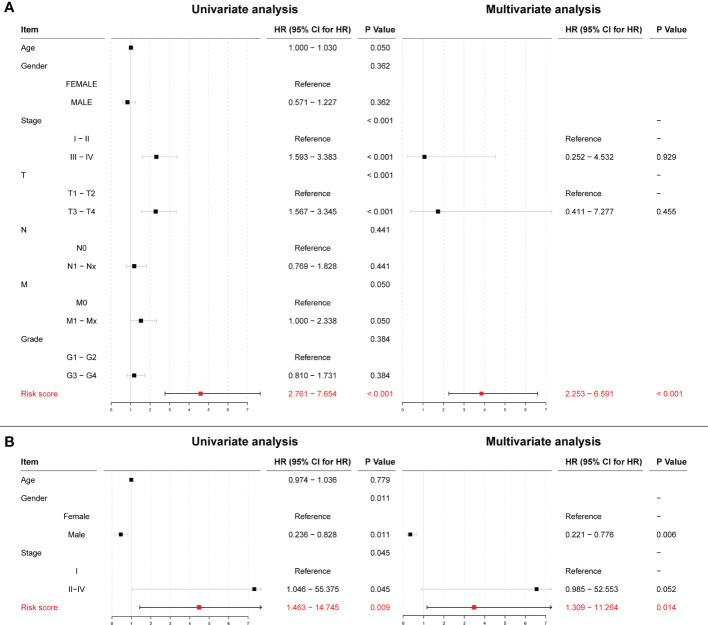
**(A)** Univariate and multivariate Cox regression results for risk scores in the TCGA cohort. **(B)** Univariate and multivariate Cox regression results for risk scores in the ICGC cohort.

After scoring each patient according to the prognostic model, the patients were divided into high-risk and low-risk groups according to the median score. The results showed statistically significant differences in OS, RFS, and DFS between patients in the high-risk and low-risk groups (p < 0.0001, p = 0.0021, and p < 0.0001, respectively; **Figures 14C–E**). The predictive ability of the model was assessed using time-dependent ROC curves, and the AUC was 0.654, 0.717, and 0.718 at 1, 3, and 5 years, respectively (**Figure 14F**). Univariate and multivariate Cox regression results also showed that the risk score was an independent prognostic factor (p < 0.001; **Figure 15A**).

To further validate the effect of this model, we performed an external validation using ICGC data. The results again showed a statistically significant difference in OS between patients in the high- and low-risk groups (p = 0.0086; **Figure 14G**). The time-dependent ROC curves had 1-, 3-, and 5-year AUCs of 0.723, 0.629, and 0.718, respectively; **Figure 14H**). Univariate Cox regression results (p = 0.009) and multivariate Cox regression results (p = 0.014; **Figure 15B**) again showed that the risk score was an independent influencer of prognosis.

Finally, to explore the relationship between the risk score, prognostic model gene expression, clinicopathological parameters, and iTLS, we plotted a heatmap of gene expression in the prognostic model (**Figure 14I**). As seen in the figure, the risk score increases with higher gene expression of PITHD1, COQ3, KCNE4, SMIM3, and CCDC88B, whereas the opposite is true for CCR7, which may herald these genes as potential promising targets for immunotherapy. In addition, the figure clearly shows that the chance of iTLS emergence decreases with increasing risk scores.

## 4 Discussion

We replicated previous studies based on pathological tissue sections and survival data from patients with HCC; our findings differed slightly from that of previous studies. Previously, iTLS was thought to be associated with improved RFS in the early stages of HCC (8, 11). However, we found that this improvement is not limited to the early stages, but is reflected throughout the entire period (1, 2, and 5 years). In addition, we found a strong relationship between iTLS and improvement of DFS. However, consistent with our results, other recent studies on iTLS in HCC did not find improvement in patient OS, and controversy remains regarding the role of TLS in HCC Further studies are required to investigate and resolve this controversy. Combined with the gene expression data, we identified 20 genes that have an important relationship with iTLS formation in HCC. We demonstrated that these genes are closely related to the immune system in terms of cellular infiltration, biological functions, and signaling pathways. In addition, we found that most signature genes had some degree of sensitivity to immune checkpoint inhibitors. Considering these findings, we can speculate that signature genes may be promising targets for future HCC treatment and further demonstrate the protective effect of iTLS on HCC prognosis. However, unlike our results, most previous reports found that the number of patients in a TLS-positive group was less than that in a TLS-negative group. We speculate that these results were due to the inclusion of TIL in the TLS-positive group. In fact, there is no precise method for distinguishing between TLS and TIL, and the boundary between the two definitions is blurred. Therefore, to maximize the prognostic impact of TLS, we included TIL in the TLS-positive group, which may have led to the difference in results. Clarice et al., used the same method (17).

Our findings showed that the signature genes correlated with most of the previously reported TLS signatures, highlighting the accuracy of our signature genes. Additionally, a large number of immune-related genes were present in the signature genes, including SYTL1, ARL4D, PITHD1, CCR7, LCK, CCDC88B, CCL21, RASAL3, CXCR3, CD3D, PSTPIP1, and KCNE4. Among these, SYTL1, the gene with the highest importance in the random forest results, may play an important role in cytotoxic granule cytokinesis in lymphocytes (30, 31). There is evidence that ARL4D can control T effector function by limiting IL-2 production (32), and other genes, such as PITHD1, CCR7, LCK, and CCDC88B, have also been shown to have different effects on immunity (22, 33–36). These results corroborate the important influence of immunity on the development of HCC.

To our knowledge, we are the first to report the associations of TMEM25, COQ3, ITPRIP, DBT, and PLAU with HCC. TMEM25 has been identified as a member of the immunoglobulin superfamily, which is a target of pharmacogenomics in oncology and regenerative medicine (37). PLAU is of great importance in renal cell carcinoma (38). COQ3 has been shown to have an important role in the prognosis of esophageal cancer (39). ITPRIP has also been reported in patients with colon cancer (40). DBT has been shown to have an important effect on primary biliary cirrhosis (41).

As an immune structure, the tumor immune microenvironment to which TLS is directly exposed is important for its formation. We explored TLS signature genes within HCC tumors from the perspectives of signaling pathways and immune cell infiltration using GSEA, CIBERSORT, GSCA, and GO enrichment analysis. The results of GSEA, using both the KEGG and Reactome databases, demonstrated that differentially expressed genes between TLS+ and TLS-group samples are associated with a large number of immune pathways, which can be further verified by GO analysis. The immune infiltration score showed that our signature genes are closely associated with the infiltration of immune cells, such as CD8T, Tregs, CD4T, and NK. These cells play different roles in the progression of the disease during the fight against HCC (42, 43), which also implies that the signature genes can influence the development of HCC through immunity. We found that CNV with signature genes, regardless of type, caused a decrease in immune infiltration, which may demonstrate that signature genes have an important role in immune cell recruitment. Understanding gene mutations in different samples and exploring the upstream and downstream signaling pathways affected by mutated genes is important for the development of targeted cancer therapies. We discovered that most of the mutations in iTLS-related signature genes were concentrated in the RTK-RAS pathway, and that overactivation of this pathway was closely related to HCC (44). After exploring the drug sensitivity of the signature genes, we found that RASAL3 and CORO1A had strong sensitivity to most of the drugs. This finding may lead to potential improvement and/or new development of targeted therapies.

The purpose of medical research is ultimately clinical, and our prognostic model may improve the assessment of patient prognosis. More importantly, this study provides further support for the protective role of iTLS in HCC. However, for several reasons, we were unable to perform basic experiments to further validate our findings, which is one of the limitations of this study. Research of iTLS in HCC is currently at an early stage, and presently a lack of evidence is common. Therefore, more in-depth study is urgently needed to demonstrate the effect of iTLS on HCC. iTLS’ positive effect in HCC has been repeatedly reported and was validated in this study. However, additional large multicenter studies and emphasis on the importance of continued investment in research are required to elucidate the specific functional mechanisms of iTLS. A key issue to consider before conducting further studies is to unify the evaluation criteria for TLS. Currently, scholars use different criteria to define TLS, which inevitably causes errors in research results. Predictably, in the near future, the development of artificial intelligence and improved computer technology will make standardization of TLS identification possible. With improved standardization, TLS will be a promising tool to add to the arsenal in the clinical fight against cancer.

## 5 Conclusion

We found that the improvement of RFS in patients with HCC due to iTLS is not limited to the early period as previously reported but is reflected throughout the entire period. In addition, we found that iTLS could improve DFS. Combined with the gene expression data, we identified 20 genes that have an important relationship with iTLS formation in HCC. We demonstrated that these genes are closely related to immunity in terms of cellular infiltration, biological functions, and signaling pathways. In addition, we found that the majority of the signature genes had some degree of sensitivity to immune checkpoint inhibitors. Considering these findings, we speculate that signature genes may be promising targets for future HCC therapy and further demonstrate the protective effect of iTLS on HCC prognosis.

## Data availability statement

The original contributions presented in the study are included in the article/**Supplementary Material**. Further inquiries can be directed to the corresponding authors.

## Ethics statement

Ethical review and approval was not required for the study on human participants in accordance with the local legislation and institutional requirements. Written informed consent for participation was not required for this study in accordance with the national legislation and the institutional requirements.

## Author contributions

WJ contributed to the concept and design of the study. QY carefully reviewed the first draft of the article. TZ, JL, YN, WS, and XL participated in the discussions. WJS oversaw all aspects of literature review design and manuscript writing. All authors contributed to the manuscript and approved the submitted version.

## Funding

This study was supported by the National Natural Science Foundation of China (81672716).

## Conflict of interest

The authors declare that the research was conducted in the absence of any commercial or financial relationships that could be construed as a potential conflict of interest.

## Publisher’s note

All claims expressed in this article are solely those of the authors and do not necessarily represent those of their affiliated organizations, or those of the publisher, the editors and the reviewers. Any product that may be evaluated in this article, or claim that may be made by its manufacturer, is not guaranteed or endorsed by the publisher.
