# Supplementary material for: Protective effect of tertiary lymphoid structures against hepatocellular carcinoma: New findings from a genetic perspective
Source: Front Immunol. 2022 Sep 14;13:1007426. doi: 10.3389/fimmu.2022.1007426 (PMC9515394; doi:10.3389/fimmu.2022.1007426)
Supplement: Supplementary file 1 [file Image_1.pdf]

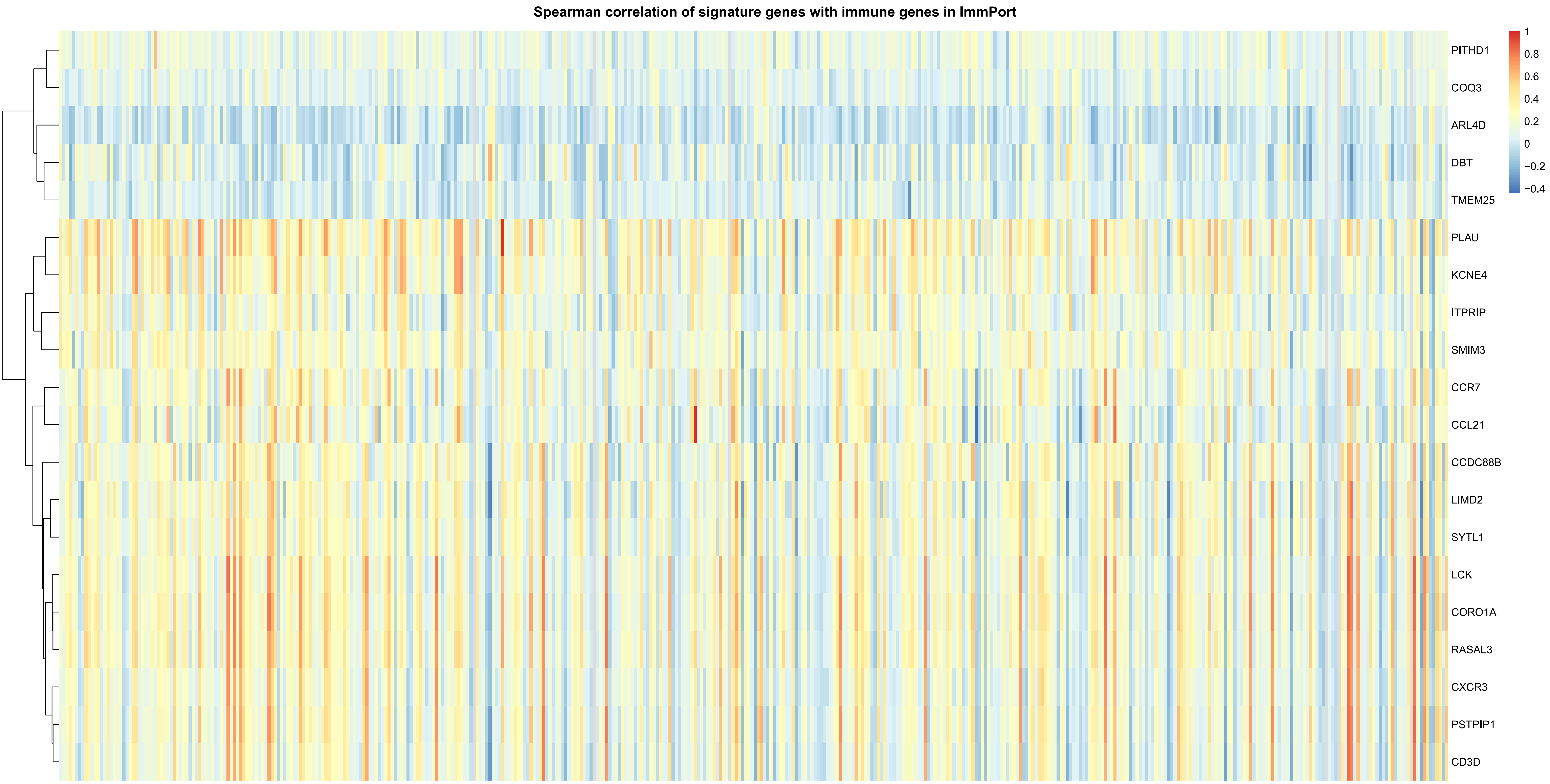

The rows are signature genes and the columns are immune-related genes in ImmPort. Note that the p-values cannot be shown in the figure due to the size of the image, and the color of the block indicates the strength of the correlation. p-values and spearman correlations are shown in the supplementary table.
